# Myocardial Infarction-Associated Shock: A Comprehensive Analysis of Phenotypes, SCAI Classification, and Outcome Assessment

**DOI:** 10.3390/medicina61010103

**Published:** 2025-01-13

**Authors:** Stanislav Dil, Maria Kercheva, Oleg Panteleev, Sergey Demianov, Aleksandr Kanev, Nina Belich, Boris Kornienko, Vyacheslav Ryabov

**Affiliations:** 1 Cardiology Research Institute, Tomsk National Research Medical Center, Russian Academy of Sciences, Tomsk 119334, Russia; kercheva@cardio-tomsk.ru (M.K.); reverso@mail.ru (B.K.); rvvt@cardio-tomsk.ru (V.R.); 2 Cardiology Division, Siberian State Medical University, 2 Moscovsky Trakt, Tomsk 634055, Russia

**Keywords:** myocardial infarction-associated shock, mixed shock, cardiogenic shock, systemic inflammatory response syndrome

## Abstract

*Background and Objectives*: In-hospital mortality associated with myocardial infarction complicated by cardiogenic shock (MI-CS) remains critically high. A particularly challenging form, mixed shock (MS), combines features of cardiogenic shock (CS) with distributive elements such as vasodilation and reduced vascular resistance. MS is associated with elevated mortality rates and presents unique diagnostic and therapeutic challenges. This study aimed to analyze the clinical, historical, instrumental, and laboratory characteristics of the primary phenotypes of MI-CS, stratified according to the Society for Cardiovascular Angiography and Interventions (SCAI) shock severity scale. *Materials and Methods*: In this single-center, retrospective observational study, we reviewed the medical records of 1289 patients admitted to the emergency cardiology department from 1 January to 12 December 2020. Among them, 117 patients were identified as having MI-CS and were divided into two groups: MS (*n* = 48) and isolated CS (*n* = 69). The data were analyzed using the SCAI shock classification. Logistic regression analysis was employed to identify predictors of mortality and improved survival outcomes. *Results*: Patients with MS were older (80 years [71.0; 83.0] vs. 73 years [64.0; 81.0], *p* = 0.035). The overall mortality rate was significantly higher in the MS group (68% vs. 53%, RR = 1.438, 95% CI: 1.041–1.986, *p* = 0.03). Logistic regression identified mechanical ventilation (OR = 8.33, 95% CI: 2.54–22.80, *p* = 0.012), elevated lactate levels (OR = 1.20, 95% CI: 1.02–1.41, *p* = 0.026), and cardiopulmonary resuscitation (CPR) (OR = 7.97, 95% CI: 2.51–24.40, *p* < 0.0001) as independent predictors of mortality. Conversely, the use of an intra-aortic balloon pump (IABP) (OR = 0.22, 95% CI: 0.06–0.80, *p* = 0.021) and a higher body mass index (BMI) (OR = 0.91, 95% CI: 0.84–0.99, *p* = 0.038) were associated with reduced mortality risk. *Conclusions*: MS in the context of MI-CS represents a distinct clinical phenotype with specific hemodynamic features and significantly worsened outcomes. The identification of mortality predictors, such as mechanical ventilation, elevated lactate levels, and CPR, alongside protective factors like IABP use and a higher BMI, underscores the importance of early and tailored therapeutic interventions. These findings highlight the need for further studies to refine treatment protocols and improve outcomes in this high-risk population.

## 1. Introduction

Cardiogenic shock (CS) represents a state of critical organ hypoperfusion and hypoxia resulting from primary cardiac dysfunction [1]. This life-threatening condition, characterized by inadequate tissue perfusion, is a major cause of morbidity and mortality worldwide, particularly in the context of acute myocardial infarction (MI). MI is the cause of CS in 80% of cases and is one of the most challenging and life-threatening clinical scenarios in cardiology [2]. The efficacy of early myocardial revascularization and contemporary therapies aimed at supporting cardiac pump function in this patient group is well established; however, in-hospital mortality among patients with MI-associated shock (MI-CS) remains extremely high [3,4,5,6,7]. Despite advances in therapeutic interventions, the management of MI-CS remains a critical challenge due to its complex pathophysiology and the need for timely intervention. Without timely specialized medical intervention, mortality rates can reach 90% [8].

Mixed shock (MS) is a combination of CS with features of another shock type (distributive, hypovolemic) [9]. The presence of mixed shock complicates the management of patients, as the hemodynamic profile can be more difficult to interpret and treat effectively. Some researchers define MS as CS with a hemodynamic profile characterized by a low cardiac output, elevated filling pressures, and low systemic vascular resistance [1]. More than a quarter of patients admitted with shock can be classified as having MS [10,11,12]. The most common cause of MS is a combination of CS and septic shock (CS with a “distributive-inflammatory component”) [12,13]. This type of shock is associated with a more complex inflammatory response, which exacerbates tissue damage and worsens clinical outcomes. This phenotype of MI-CS is characterized by signs of systemic inflammatory response syndrome (SIRS), lower systemic vascular resistance, and a higher risk of sepsis and mortality [14,15]. Recent studies, including those assessing outcomes with veno-arterial extracorporeal membrane oxygenation (ECMO), have shown that mortality rates in MS cases can surpass those in isolated CS cases, reaching 68% compared to 53% for CS alone [13].

Despite its prevalence and impact, MS in the context of MI remains underexplored, especially regarding the contribution of SIRS and other pathogenic factors to poor outcomes. The ability to accurately distinguish MI-CS phenotypes upon admission could be pivotal in optimizing therapeutic strategies, as each phenotype may require a tailored approach to address its specific pathophysiology [16,17]. The SCAI (Society for Cardiovascular Angiography and Interventions) scale [18], originally developed to categorize CS severity, is particularly relevant in this context. This scale allows for a detailed classification of shock based on clinical trajectory and response to treatment, which may provide more accurate prognostication than general ICU severity scales like SOFA [19,20]. This study uniquely focuses on the mixed shock phenotype in myocardial infarction, a condition that remains underexplored despite its significant clinical impact.

The aim of this study is to identify and characterize the primary phenotypes of myocardial infarction complicated by MI-CS, focusing on their clinical, historical, instrumental, and laboratory profiles to improve prognostication and guide tailored therapeutic strategies.

## 2. Materials and Methods

### 2.1. Study Design

This single-center, observational, and retrospective study was conducted at the Cardiology Research Institute, part of the Tomsk National Research Medical Center, and was approved by the local ethics committee. All methods and procedures adhered to the ethical principles outlined in the Declaration of Helsinki. No cases of withdrawal of consent were documented.

We reviewed 1289 medical records of patients admitted to the emergency cardiology department from 1 January 2020 to 12 December 2020. The study cohort included patients diagnosed with MI-CS within 24 h of hospitalization, according to the International Classification of Diseases, 10th Revision (ICD-10). Patients were subdivided into two groups: Group 1 consisted of those with MI-CS combined with either distributive or hypovolemic shock (termed mixed shock, MS), and Group 2 included patients with isolated CS.

Myocardial infarction was classified according to the Fourth Universal Definition of MI [21], with confirmation of ST-Elevation Myocardial Infarction (STEMI) or Non-ST-Elevation Myocardial Infarction (NSTEMI) status. Reperfusion delays were defined as the time from first medical contact to the onset of reperfusion therapy, including percutaneous coronary intervention (PCI), where applicable.

We defined CS for the purposes of patient inclusion based on established clinical criteria. CS was characterized by persistent hypotension (systolic blood pressure < 90 mm Hg or the need for vasopressors to maintain blood pressure) and evidence of end-organ hypoperfusion (such as altered mental status, cool extremities, oliguria, or elevated lactate levels) [22].

Patients were assigned to the MS group based on clearly defined criteria. First, persistent hypotension unresponsive to fluid resuscitation was required, with patients needing vasopressors to maintain a mean arterial pressure above 65 mmHg. Additionally, evidence of possible infection was necessary, indicated by at least one of the following: fever, leukocytosis, elevated inflammatory markers (such as C-reactive protein or procalcitonin), or clinical signs suggestive of infection (e.g., respiratory symptoms or suspected pneumonia). To further differentiate distributive shock components, echocardiography was used to identify at least one hemodynamic marker of distributive shock, such as hyperdynamic left ventricular (LV) function with reduced systemic vascular resistance, low left atrial filling pressure (e.g., <10 mmHg), a high cardiac index (e.g., >3.5 L/min/m²) combined with systemic vasodilation, and dilated inferior vena cava with respiratory variation suggesting low right atrial pressure. These criteria ensured that patients in the MS group exhibited both cardiogenic and distributive shock features [23,24,25].

Hemorrhagic (hypovolemic) shock was defined by significant blood loss leading to hemodynamic instability despite fluid resuscitation of at least 500 mL, with clinical signs of organ hypoperfusion and estimated blood loss >40% [26].

Bleeding sources were identified using endoscopic methods (endoscopy). We categorized hemorrhages into upper gastrointestinal (e.g., peptic ulcers) and distal gastrointestinal bleeding, recording the interventions applied (pharmacological therapy, blood transfusion, or surgical intervention).

Shock severity was retrospectively classified according to the Society for Cardiovascular Angiography and Interventions (SCAI) shock stages (A: at risk of cardiogenic shock; B: beginning shock; C: classic shock; D: deterioration; and E: extremis) at the time of admission [22]. Initial classification was based on presenting clinical and hemodynamic parameters. If a patient’s condition worsened during hospitalization, shock stage was re-evaluated, and each patient was ultimately assigned the highest (most severe) stage reached during their hospital stay. This approach ensured that classification accurately reflected the peak severity of CS experienced by each patient, aligning with SCAI’s criteria for staging dynamic clinical trajectories.

### 2.2. Clinical, Anamnestic, Laboratory, and Instrumental Data

Patient data were retrospectively collected, including clinical, anamnestic, laboratory, and instrumental findings at admission and throughout hospitalization. All patients provided informed consent for the processing of their medical data upon admission to the hospital, in accordance with institutional policies. The ethics committee subsequently authorized the retrospective use of these data for the study. No cases of withdrawal of consent were documented, and no patients were contacted post-discharge to opt out of the study due to its retrospective nature.

Patient histories were gathered primarily from medical records; when necessary, information was obtained from relatives, emergency personnel, or supplemented upon patient stabilization. The retrospective nature of the study allowed for the use of these sources to reconstruct patient histories.

Laboratory data encompassed complete blood counts, blood biochemistry, acid–base balance parameters, and blood lactate levels. Instrumental evaluations included electrocardiography, echocardiography, chest X-ray, and details of the PCI procedure, including door-to-balloon time. Diary entries of attending physicians were analyzed to cross-reference patients’ clinical status with laboratory and instrumental findings, ensuring data accuracy.

### 2.3. Statistical Analysis Methods

The primary endpoint was in-hospital mortality from all causes. The normality of the distribution of quantitative variables was assessed using the Shapiro–Wilk test. Normally distributed variables are presented as the mean and standard deviation (m ± SD), while non-normally distributed variables are presented as the median (Me) and interquartile range (Q1; Q3). Categorical variables are described by absolute and relative frequencies (%). The Mann–Whitney U test was used to identify statistically significant differences in quantitative variables between independent groups with a non-normal distribution. Student’s t-test was employed for groups with normally distributed quantitative variables. Categorical variables in independent groups were compared using the Pearson chi-square test or Fisher’s exact test. A *p*-value of 0.05 was chosen as the threshold for statistical significance.

Logistic regression analysis was conducted to identify independent predictors of mortality. Initially, a wide range of clinical and laboratory parameters was assessed; however, only those variables that demonstrated statistical significance in univariate analysis (*p* < 0.05) were included in the final models. The selected covariates included mechanical ventilation (MV), lactate levels, and cardiopulmonary resuscitation (CPR), which were associated with a higher risk of mortality, as well as intra-aortic balloon pump (IABP) use and body mass index (BMI), which demonstrated protective effects against mortality. These variables represent key factors linked to hemodynamic stability, metabolic disturbances, and acute clinical deterioration in patients with cardiogenic shock. Odds ratios (ORs) with 95% confidence intervals (CIs) were calculated to quantify the strength of associations. Model quality was evaluated using the Akaike information criterion (AIC) and receiver operating characteristic (ROC) curve analysis, while relative risks (RRs) with corresponding CIs were used to compare mortality rates across subgroups.

## 3. Results

### 3.1. Patient Population and Baseline Characteristics

**Figure 1:**
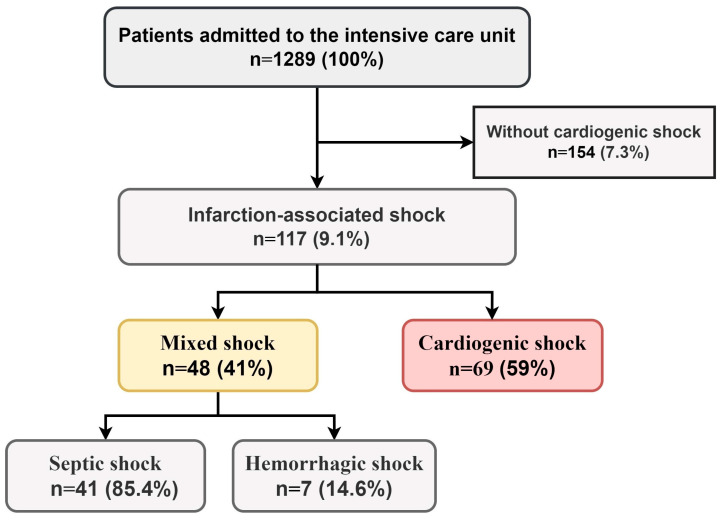
Prevalence of MI-CS phenotypes among patients admitted to intensive care unit.

From 1289 records, 117 (9.1%) patients with MI complicated by CS at admission were included in the final study sample (Figure 1). Among them, 48 (41%) exhibited a mixed shock (MS) phenotype, while 69 (59%) had isolated CS. Septic shock was diagnosed in 41 MS patients (85%), and hemorrhagic shock in 7 (15%) (Figure 1).

The MS group was older than the CS group (80 [71; 83] vs. 73 [64; 81] years, *p* = 0.035). Hypertension was less frequent in MS patients than in CS patients (88% vs. 97%, *p* = 0.045). Other historical data were not significantly different between groups (Table 1).

### 3.2. Characteristics of Acute Myocardial Infarction and PCI Parameters

There were no significant differences in baseline MI characteristics, including infarction phenotype, location, or infarct-related coronary artery (IRCA). The door-to-balloon time was similar between groups (73 [50; 116] minutes vs. 67 [51; 121] minutes, *p* = 0.616). TIMI 3 flow restoration in the IRCA was achieved in 46% of MS patients and 51% of CS patients (*p* = 0.786). MI complications and PCI rates were comparable between groups (Table 2).

The complications observed included repeat PCI, recurrent MI, mechanical complications of MI, and the formation of left ventricular aneurysms. Specifically, repeat PCI was performed in 8 (17%) of the MS group and in 13 (19%) of the CS group (*p* = 0.842). Recurrent MI occurred in 10 (21%) of the MS group and in 11 (16%) of the CS group (*p* = 0.521). Mechanical complications of MI, such as ventricular septal rupture and free wall rupture, were noted in 4 (8.3%) of the MS group and 10 (15%) of the CS group (*p* = 0.315). Left ventricular aneurysms were identified in 8 (17%) of the MS group and 15 (22%) of the CS group (*p* = 0.507).

### 3.3. Laboratory and Instrumental Data and Risk Stratification

The GRACE (Global Registry of Acute Coronary Events) score for in-hospital mortality and the CRUSADE (Can Rapid risk stratification of Unstable angina patients Suppress ADverse outcomes with Early implementation of the ACC/AHA guidelines) score for major bleeding were significantly higher in MS patients than in CS patients (49 [28; 61]% vs. 26 [11; 50]% and 19 [13; 20]% vs. 12 [8.6; 20]%, respectively, *p* < 0.001). Other scores, such as GENEVA, ORBI, and SOFA, showed no significant group differences (Table 3).

Due to limited sample sizes, SCAI stages A and B (A + B) were combined, as were stages D and E (D + E), for statistical analysis. The MS group showed a lower frequency of stage C shock compared to the CS group (52% vs. 70%, *p* = 0.001), while stages D + E were more frequent in the MS group than in the CS group (46% vs. 20%, *p* < 0.001) (Table 3).

Patients in the MS group had a significantly lower blood pH (7.25 [7.17; 7.32] vs. 7.31 [7.26; 7.34], *p* = 0.017) and higher blood lactate levels (5.8 [3.0; 7.7] mmol/L vs. 3.5 [2.4; 6.2] mmol/L, *p* = 0.015). Additionally, MS patients had lower total protein levels (64 [58; 67] g/L vs. 67 [60; 75] g/L, *p* = 0.026) and lower glomerular filtration rates (36 [25; 51] vs. 43 [29; 63] mL/min/1.73 m², *p* = 0.004). No other significant laboratory differences were identified (Table 3).

Baseline echocardiographic data, including parameters of cardiac function, did not significantly differ between groups (Table 3).

Patients in both groups received antithrombotic, cardioprotective, diuretic, and hypolipidemic therapies without intergroup differences (Table 4).

### 3.4. Treatment

Mechanical ventilation (MV) was more frequent (90% vs. 71%, *p* = 0.006) and of longer duration (4 [1; 9] vs. 2 [1; 4] days, *p* = 0.001) in MS patients. The MS group had higher rates of blood transfusion (38% vs. 8.7%, *p* < 0.001). Six out of seven patients with hemorrhagic shock experienced upper gastrointestinal bleeding, predominantly from peptic ulcers, while one patient had distal gastrointestinal bleeding. Hemostatic interventions were successful in six patients, while one case resulted in fatal outcomes. PCI was performed in 75% of MS patients and 64% of CS patients (*p* = 0.393). There was a trend toward increased use of an intra-aortic balloon pump (IABP) in MS patients (31% vs. 19%, *p* = 0.150) with longer durations (68 [26; 134] vs. 32 [18; 47] hours, *p* = 0.058). No differences in hospital stay duration or ICU time were observed (Table 4).

The MS group had a higher prevalence of pneumonia compared to the CS group (50% vs. 29%, *p* < 0.001), particularly ventilator-associated pneumonia (38% vs. 25%, *p* = 0.008). Mortality associated with pneumonia was 56%.

### 3.5. Mortality

The overall mortality rate in the MS group was significantly higher than in the CS group (68% vs. 53%, RR = 1.438, 95% CI: 1.041–1.986, *p* = 0.03).

### 3.6. Risk Factors for Mortality

Logistic regression identified several independent predictors of mortality. Mechanical ventilation (OR = 8.33, 95% CI: 2.54–22.80, *p* = 0.012), elevated lactate levels (OR = 1.20, 95% CI: 1.02–1.41, *p* = 0.026), and CPR (OR = 7.97, 95% CI: 2.51–24.40, *p* < 0.0001) were associated with a higher risk of death. Conversely, the use of an IABP (OR = 0.22, 95% CI: 0.06–0.80, *p* = 0.021) and a higher BMI (OR = 0.91, 95% CI: 0.84–0.99, *p* = 0.038) demonstrated protective effects against mortality.

The quality of model fit for the logistic regression was assessed using McFadden’s pseudo-R^2^. The pseudo-R^2^ value was 0.5, indicating a moderate improvement in the model’s predictive ability compared to the null model.

The performance of the constructed model was evaluated through ROC analysis, including the calculation of sensitivity, specificity, and the area under the ROC curve (AUC). The predictive accuracy of the model was validated on a test dataset, yielding a sensitivity of 86.3%, a specificity of 92.9%, an accuracy of 90.6%, and an AUC of 0.97.

## 4. Discussion

The results of our study, alongside existing scientific evidence, emphasize that patients with MS represent a high-risk group that remains insufficiently studied [10,12,27]. MS is characterized by a combination of CS with features of another type of shock, most commonly septic shock. While hemorrhagic shock is less frequent in MS, it poses a significant challenge due to its association with higher transfusion rates and mortality. Our findings align with prior evidence that hemorrhagic complications in MI-CS are associated with a poor prognosis. Including patients with this phenotype broadens our understanding of MS and reflects real-world clinical scenarios.

While our study acknowledges the foundational work by Berg et al. (2019), it provides a novel perspective by focusing on the mixed shock phenotype within MI-CS, an area that remains underexplored [10].

Our data confirm that patients with MS have significantly higher mortality rates com-pared to those with isolated CS (68% vs. 53%, RR = 1.438, 95% CI: 1.041–1.986, *p* = 0.03). This highlights the urgent need for improved management strategies tailored to this complex phenotype. Logistic regression identified several independent predictors of mortality, including MV, elevated lactate levels, and the need for CPR. These variables were strongly associated with an increased risk of death and underscore the importance of close monitoring of these parameters for early identification of high-risk patients. Specifically, the use of MV was associated with an 8-fold increase in mortality risk (OR = 8.33, 95% CI: 2.54–22.80, *p* = 0.012), reflecting the severity of respiratory failure in these patients. Elevated lactate levels (OR = 1.20, 95% CI: 1.02–1.41, *p* = 0.026) were indicative of metabolic dysfunction and tissue hypoperfusion, further emphasizing the need for careful monitoring of this marker during treatment.

In contrast, the use of an IABP and a higher BMI were associated with reduced mortality. This aligns with the existing literature suggesting that an IABP can improve hemodynamic stability in patients with cardiogenic shock, although its effectiveness appears limited in MS. While an IABP has been shown to significantly improve outcomes in isolated CS, its benefit in MS patients may be attenuated due to the underlying complex pathophysiology [28].

BMI, typically considered a risk factor for various cardiovascular diseases, in our cohort showed a protective effect (OR = 0.91, 95% CI: 0.84–0.99, *p* = 0.038). This finding may seem counterintuitive, as BMI is generally considered a risk factor for various cardiovascular diseases. However, in this context, it aligns with the existing literature suggesting a phenomenon known as “the obesity paradox”. This paradox has been well documented in studies involving patients with heart failure, acute myocardial infarction, and sepsis. The protective effect of obesity in these acute settings may be explained by several mechanisms. One hypothesis is that excess adipose tissue serves as a metabolic reserve, providing a buffer against acute metabolic stresses such as hypoxia, inflammation, and nutritional deficits. Additionally, increased adiposity may contribute to better hemodynamic stability, improving the ability to tolerate the physiological demands of acute shock [29,30]. The hemodynamic profile of MS is characterized by a combination of a reduced cardiac output and low systemic vascular resistance. Although systemic vascular resistance varies widely in MI-CS patients, it is generally close to normal and often artificially elevated by vasopressors [31,32].

A notable observation was the relatively preserved cardiac pump function, as assessed by echocardiography, in both groups. This diverges from the traditional view that acute left ventricular systolic dysfunction is the primary driver of cardiogenic shock. While diminished cardiac pump function remains critical in MI-CS, additional factors such as systemic inflammatory response syndrome (SIRS) and mitochondrial dysfunction appear to exacerbate the condition. Elevated lactate levels in MS patients reflect profound metabolic impairment, or “cellular dysoxia”, which encompasses not only oxygen delivery deficits but also disrupted oxygen utilization at the cellular level [33].

Our study also highlights the multifactorial nature of MI-CS pathophysiology. For instance, despite the stabilizing effects of mechanical circulatory support (MCS) devices on cardiac output, clinical outcomes often remain suboptimal [34,35,36,37]. This suggests that MCS may not adequately address peripheral perfusion deficits or cellular dysoxia. Therefore, comprehensive treatment approaches targeting both cardiac and peripheral pathologies—particularly inflammatory processes and metabolic dysfunction—are essential for improving outcomes in MI-CS.

Inflammatory processes, driven by myocardial necrosis and cytokine release, play a pivotal role in worsening shock and organ dysfunction in MI-CS patients. Cytokine storms induced by factors such as interleukins, tumor necrosis factor, and inducible nitric oxide synthase contribute to systemic vasodilation and the progression to distributive shock, even in the absence of overt infection [1,27,38,39].

In our cohort, non-invasive hemodynamic assessment combined with clinical infection indicators (e.g., fever, elevated inflammatory markers) allowed for the identification of a distributive hemodynamic profile. This profile, coupled with higher requirements for MV and longer MV durations in MS patients, likely reflects a combination of ARDS secondary to pneumonia and cardiogenic pulmonary edema. While sympathomimetic infusion rates were similar across the groups, MS patients may have required additional fluid resuscitation to offset vasoplegia, a strategy that could be more limited in isolated MI-CS cases.

These findings suggest that the presence of a distributive hemodynamic profile in MI-CS, identified via non-invasive methods, should be considered a likely indicator of SIRS progression and the development of MS. Such early identification could expedite the use of efferent therapies aimed at mitigating key SIRS-related pathogenic components, such as the cytokine storm, potentially pre-empting shock progression to SCAI stages D and E, where mortality rates can approach 90% [40]. Our previous findings further support the efficacy of hemoadsorption therapy in managing stage D MS, emphasizing the importance of early intervention [41].

Our findings confirm that MS patients face a significantly higher risk of adverse outcomes despite aggressive interventions, such as more frequent MV, blood transfusions, and prolonged IABP use. These results align with previous studies showing that MS is associated with an extremely poor prognosis, even with intensive management [10,12]. In previous research on this cohort [42], we found no correlation between MI-CS severity or outcomes and MI characteristics, with tissue and organ hypoperfusion intensifying across SCAI stages, as evidenced by decreasing central venous blood pH.

The prognostic utility of the SCAI classification was reaffirmed, with MS patients more frequently classified as stages D and E, correlating with higher severity and worse outcomes. In contrast, the SOFA scale failed to adequately capture these distinctions, underscoring the SCAI scale’s value in guiding treatment planning for diverse shock phenotypes [20].

Overall, our findings emphasize the need for phenotyping MS patients who experience severe disease progression, high mortality rates, and an increased requirement for intensive therapies. Our results also suggest that SIRS and its associated cellular and molecular effects may play a central role in MS pathogenesis. Future research should focus on identifying novel therapeutic targets to address these complex pathophysiological mechanisms

### 4.1. Novelty and Practical Significance of Study

This study represents one of the most comprehensive comparative analyses of patients with distinct MI-CS phenotypes to date. By integrating clinical, anamnestic, instrumental, and laboratory characteristics with regression analysis, we identified key predictors of adverse outcomes and mortality, offering valuable insights for refining prognostic models and risk stratification criteria in cardiogenic shock.

This study provides a robust foundation for the development of tailored treatment protocols, enabling clinicians to address the multifactorial pathogenesis of MI-CS more effectively. By advancing our understanding of these phenotypes, it paves the way for improved clinical decision-making and better outcomes for this high-risk population in cardiac intensive care.

### 4.2. Study Limitations

The primary limitations of our study are its retrospective design and single-center nature, which may constrain the generalizability of our findings to broader populations. The relatively small sample size, particularly when stratifying patients across different SCAI shock stages, poses additional limitations, potentially reducing the statistical power of subgroup analyses. Furthermore, the heterogeneity within the mixed shock (MS) group reflects the inherent complexity of this clinical condition, which combines overlapping pathophysiological mechanisms, including cardiogenic, distributive, and hypovolemic components. While this diversity complicates direct comparisons, it mirrors real-world scenarios and underscores the importance of individualized treatment strategies for these patients.

Another limitation stems from the brevity of hospital stays in some patients, particularly those with high early mortality. This precluded a comprehensive diagnostic assessment, including invasive hemodynamic monitoring, extended echocardiographic evaluations, and detailed laboratory analyses. Similarly, while septic shock was a frequent component of MS, the availability of microbiological data, such as blood culture results or the precise microbial etiology, was limited in many cases. In patients with hemorrhagic shock, details regarding the source of bleeding and management strategies were collected retrospectively and may not fully capture the dynamic clinical context.

Despite these limitations, our study offers valuable insights into a poorly understood population. By examining the interaction of cardiogenic and non-cardiogenic shock components, we provide a foundation for future studies to stratify MS patients based on their predominant shock phenotype. Detailed investigations into infectious and hemorrhagic processes—such as microbial etiology, antibiotic regimens, and hemostatic interventions—will help refine our understanding of MS pathophysiology and improve treatment outcomes. Moreover, prospective multicenter studies with larger sample sizes and more extensive diagnostic protocols will be essential to validate and extend our findings.

## 5. Conclusions

Phenotyping MI-CS remains a complex challenge due to the heterogeneity of prognostic indicators, with no single tool offering sufficient accuracy. However, the importance of precise phenotyping cannot be overstated, as it is crucial for the development of more targeted and effective therapies. The distributive hemodynamic phenotype, marked by vasodilation and reduced vascular resistance, may signal early involvement of mixed shock in MI-CS. Our data suggest that MS patients, who are typically older and have more comorbidities, experience more severe shock stages, higher mortality, and worse outcomes compared to those with isolated cardiogenic shock, regardless of MI severity. Infectious comorbidities notably worsen outcomes in MI-CS, complicating both diagnosis and treatment. Additionally, the potential protective roles of factors such as BMI and IABPs warrant further investigation, including at the molecular and cellular levels, so we can better understand their mechanisms and refine early targeted therapies for improved patient outcomes.

## Figures and Tables

**Table 1:** Clinical and anamnestic characteristics of patients.

Variable	n	Shock Phenotype		*p*
		Mixed (n = 48)	Cardiogenic (n = 69)	
Demographic data:				
Age, years	117	80 (71.0; 83.0)	73 (64.0;81.0)	**0.035**
Female gender, n (%)	117	27 (56.0)	35 (51.0)	0.555
Risk factors and comorbidities:				
Hypertension, n (%)	117	42 (88.0)	67 (97.0)	**0.042**
Diabetes mellitus, n (%)	117	18 (38.0)	16 (23.0)	0.104
Chronic lung disease, n (%)	116	12 (25.0)	12 (17.0)	0.335
Genitourinary diseases, n (%)	117	13 (27.0)	18 (26.0)	0.904
Gastrointestinal tract disorders, n (%)	117	28 (58.0)	49 (71.0)	0.154
Oncology, n (%)	115	2 (4.3)	2 (2.9)	0.677
Stroke, n (%)	116	5 (11.0)	12 (17.0)	0.424
Smoking, n (%)	79	11 (28.0)	18 (38.0)	0.349
Alcohol use, n (%)	84	0 (0)	6 (12.0)	0.076
Objective and instrumental data:				
Glasgow coma scale, points	113	12 (9.0; 14.0)	14 (10.0; 15.0)	0.084
Systolic blood pressure, mm Hg	117	80 (70.0; 90.0)	94 (78.0; 122.0)	**<0.001**
Mean arterial pressure, mm Hg	117	60.0 ± 16.0	72.0 ± 23.0	**0.002**
Heart rate, bpm	117	90 (56.0; 111.0)	89 (73.0; 106.0)	0.735
Respiratory rate, breaths/min	108	20 (17.0; 24.0)	18 (16.0; 22.0)	0.130
Oxygen saturation (SpO_2_), %	94	93 (90.0; 96.0)	94 (90.0; 96.0)	0.594
Central venous pressure, cm H_2_O	85	13 (7.5; 16.0)	12 (8.0; 16.0)	0.597

Variables are presented as the mean and standard deviation (m ± SD) when they are normally distributed, and as the median (Me) and interquartile range (Q1; Q3) otherwise. Categorical variables were described by absolute and relative frequencies (%). Boldface denotes statistical significance.

**Table 2:** Characteristics of acute myocardial infarction course and PCI parameters.

Variable	n	Shock Phenotype		*p*
		Mixed (n = 48)	Cardiogenic (n = 69)	
Myocardial Infarction Characteristics:				
STEMI, n (%)	117	39 (81.0)	52 (75.0)	0.451
NSTEMI, n (%)	117	9 (19.0)	17 (25.0)	0.150
Primary MI, n (%)	117	28 (58.0)	38 (55.0)	0.726
PCI, n (%)	117	33 (75.0)	43 (64.0)	0.393
Door-to-balloon time, minutes	117	73 (50.0; 16.0)	67 (51.0;121.0)	0.616
TIMI 3 flow in PCI, n (%)	117	22 (46.0)	35 (51.0)	0.786
MI Localization:				
Anterior, n (%)	117	24 (50.0)	42 (61.0)	0.244
Inferior, n (%)	117	24 (50.0)	27 (39.0)	0.244
IRCA:				
LAD, n (%)	76	19 (40.0)	20 (29.0)	0.079
RCA, n (%)	76	14 (29.0)	16 (23.0)	0.330
DA, n (%)	76	0 (0)	3 (4.4)	0.665
LCX, n (%)	76	8 (17.0)	5 (7.3)	0.089
OM, n (%)	76	2 (4.2)	2 (2.9)	0.917
LCA, n (%)	76	1 (2.1)	3 (4.4)	0.353
PDA, n (%)	76	1 (2.1)	3 (4.4)	0.353
Complications:				
Repeat PCI, n (%)	117	8 (17.0)	13 (19.0)	0.842
Recurrent MI, n (%)	117	10 (21.0)	11 (16.0)	0.521
Mechanical complications of MI, n (%)	117	4 (8.3)	10 (15.0)	0.315
Left ventricular aneurysm, n (%)	117	8 (17.0)	15 (22.0)	0.507

Note: IRCA—Infarct-Related Coronary Artery; LAD—left anterior descending artery; LCA—left coronary artery; LCX—circumflex coronary artery; MI—myocardial infarction; NSTEMI—Non-ST-Segment Elevation Myocardial Infarction; OM—obtuse marginal; PDA—posterior descending artery; PCI—percutaneous coronary intervention; RCA—right coronary artery; STEMI—ST-Segment Elevation Myocardial Infarction; TIMI—Thrombolysis In Myocardial Infarction.

**Table 3:** Laboratory and instrumental data and risk stratification.

Variable	n	Shock Phenotype		*p*
		Mixed (n = 48)	Cardiogenic (n = 69)	
pH (venous)	95	7.25 (7.17; 7.32)	7.31 (7.26; 7.34)	**0.017**
SpO_2_, %	93	61.0 (65.0; 75.0)	69.0 (58.0; 76.0)	0.061
Troponin on admission, ng/mL	90	0.4 (0.1; 1.3)	0.5 (0.1; 3.1)	0.658
Lactate level, mmol/L	104	5.8 (3.0; 7.7)	3.5 (2.4; 6.2)	**0.015**
Platelets, ×10^12^	117	213.0 (155.0; 257.0)	236.0 (148.0; 260.0)	0.074
Red blood cells, ×10^5^	117	4.3 (3.7; 5.0)	4.5 (4.0; 5.0)	0.421
Hemoglobin, g/L	117	124.0 ± 30.0	128.0 ± 24.0	0.426
Hematocrit, %	117	37.0 ± 8.3	38.0 ± 6.3	0.505
White blood cells, ×10^9^	117	14.0 (9.9; 17.0)	13.0 (11.0; 16.0)	0.702
Creatinine, mmol/L	117	136.0 (115.0–192.0)	116.0 (91.0; 155.0)	**0.029**
GFR, mL/min/1.73 m²	117	36.0 (25.0; 51.0)	43.0 (29.0; 63.0)	**0.004**
Total protein, g/L	74	64.0 (58.0; 67.0)	67.0 (60.0; 75.0)	**0.026**
Glucose, mmol/L	116	12.0 (9.2; 17.0)	9.8 (7.3; 15.0)	0.066
Total bilirubin, µmol/L	80	15.0 (9.7; 18.0)	14.0 (10.0; 23.0)	0.740
Instrumental data:				
Body mass index, kg/m^2^	117	27.3 (24.1; 32.1)	27.2 (24.0; 31.2)	0.786
Ejection fraction, %	103	43.0 (30.0; 56.0)	45.0 (35.0; 55.0)	0.671
LV EDV, mL	78	100.0 (88.0; 136.0)	110.0 (75.0; 130.0)	0.679
LV EDV, mL	55	60.0 (41.0; 89.0)	62.0 (40.0; 76.0)	0.986
Stroke volume, mL	53	45.0 (31.0; 52.0)	39.0 (35.0; 52.0)	0.873
Left ventricular mass, g	42	219.0 (179.0; 247.0)	210.5 (174.0; 272.5)	0.888
Left ventricular mass index, g/1.73 m^2^	42	113.0 (95.0; 135.0)	112 (100.5; 143.0)	0.741
Inferior vena cava, mm	51	21.0 (16.0; 23.0)	20 (12.0; 22.0)	0.062
Left atrium, mL	53	59.0 (42.0; 94.0)	59 (42.0; 85.0)	0.897
Right atrium, mL	34	69.0 (44.0; 92.0)	58 (42.0; 80.0)	0.516
PCWP, mm Hg	43	13.0 (12.0; 18.0)	14 (11.0; 17.0)	0.769
PASP, mm Hg	48	40.0 (30.0; 55.0)	40 (35.0; 55.0)	0.881
Cardiac index, L/min/m^2^	51	2.2 (1.8; 2.5)	2 (1.7; 2.4)	0.737
WMSI	50	1.4 (1.1; 2.1)	1.8 (1.3; 2.2)	0.284
SCAI shock stages (n = 117):				
A	2	0 (0)	2 (100.0)	**<0.001**
B	8	3 (37.0)	5 (63.0)	**<0.001**
C	71	23 (31.5)	48 (68.5)	**0.001**
D	10	5 (50.0)	5 (50.0)	**<0.001**
E	26	17 (65.0)	9 (35.0)	**<0.001**
Distribution of SCAI shock stages within groups (n = 117):				
A	117	0 (0)	2 (2.9)	
B	117	3 (6.3)	5 (7.2)	
C	117	23 (47.9)	48 (69.7)	
D	117	5 (10.4)	5 (7.2)	
E	117	17 (35.4)	9 (13.0)	
A + B	117	3 (6.3)	7 (10.0)	0.792
D + E	117	22 (46.0)	14 (20.0)	**<0.001**
Risk scores:				
ORBI, score	79	17 (12.0; 22.0)	16 (11.0; 18.0)	0.168
ORBI, %	79	40 (12.0; 70.0)	28 (10.0; 46.0)	0.120
SOFA, score (baseline)	28	11 (10.0; 14.0)	10 (5.0; 12.0)	0.197
GRACE, %	117	49 (28.0; 61.0)	26 (11.0; 50.0)	**<0.001**
CRUSADE, %	117	19 (13.0; 20.0)	12 (8.6; 20.0)	**<0.001**
GENEVA, score	117	4 (1.0; 6.0)	4 (1.0; 6.0)	0.495

Note: CRUSADE—Can Rapid risk stratification of Unstable angina patients Suppress ADverse outcomes with Early implementation of the ACC/AHA guidelines; EDV—end diastolic volume; LV—left ventricle; GFR—glomerular filtration rate; GRACE—Global Registry of Acute Coronary Events; PASP—pulmonary artery systolic pressure; PCWP—pulmonary capillary wedge pressure; SOFA—Sequential Organ Failure Assessment; SpO_2_—oxygen saturation. Boldface denotes statistical significance.

**Table 4:** Treatment and outcomes.

Variable	n	Shock Phenotype		*p*
		Mixed (n = 48)	Cardiogenic (n = 69)	
Pharmacotherapy:				
Clopidogrel, n (%)	117	44 (92.0)	58 (84.0)	0.479
Ticagrelor, n (%)	117	3 (6.2)	9 (13.0)	0.254
Prasugrel, n (%)	117	1 (2.1)	2 (2.9)	0.658
Anticoagulants, n (%)	117	48 (75.0)	66 (96.0)	0.231
Diuretics, n (%)	117	33 (69.0)	42 (60.0)	0.500
ACEi/ARBs, n (%)	117	20 (42.0)	44 (64.0)	0.056
MRA, n (%)	117	21 (44.0)	34 (49.0)	0.459
Statins, n (%)	117	43 (90.0)	62 (90.0)	0.395
Intensive therapy:				
MV, n (%)	117	43 (90.0)	47 (71.0)	**0.006**
Duration of MV, days	90	4 (1.0; 9.0)	2 (1.0; 4.0)	**0.001**
IABP, n (%)	117	15 (31.0)	12 (19.0)	0.150
Duration of IABP, hours	25	68 (26.0; 134.0)	32 (18.0;47.0)	0.058
RRT, n (%)	117	6 (13.0)	5 (7,8)	0.356
Duration of RRT, days	7	3 (2.5; 3.5)	3.0 (2.0; 3.0)	0.723
Blood transfusion, n (%)	117	18 (38.0)	6 (8.7)	**<0.001**
Inotropic support:				
Inotropes, n (%)	117	37 (77.0)	44 (64.0)	0.124
Dopamine dosage, μg/kg/min	61	8 (5.0; 10.0)	6 (5.0; 8.0)	0.129
Epinephrine dosage, μg/kg/min	11	0.1 (0.1; 0.2)	0.1 (0.1; 0.2)	0.850
Norepinephrine dosage, μg/kg/min	51	0.3 (0.2; 0.8)	0.4 (0.1; 0.6)	0.399
VIS in the first 24 h	85	25 (5.0; 47.0)	10 (5.0; 40.0)	0.540
Hospitalization:				
Pneumonia, n (%)	117	24 (50.0)	20 (29.0)	**<0.001**
Community-acquired pneumonia, n (%)	44	14 (58.0)	11 (55.0)	**0.760**
Pneumonia due to SARS-CoV-19, n (%)	44	1 (4.2)	2 (10.0)	**0.100**
Hospital-acquired pneumonia associated with MV, n (%)	44	9 (38.0)	5 (25.0)	**0.004**
Hospital-acquired pneumonia not associated with MV, n (%)	44	0 (0)	2 (10.0)	**0.100**
Mortality associated with pneumonia, n (%)	44	25 (57.0)		
Outcomes:				
Days in ICU	117	4.5 (2.0; 17.0)	4 (2.0; 10.0)	0.234
Days in hospital	117	7.5 (2.0–17.0)	7.0 (2.0–14.0)	0.781
Early mortality, n (%)	117	18 (38.0)	13 (19.0)	**0.033**
In-hospital mortality, n (%)	117	32 (67.0)	32 (46.0)	**0.030**

Note: ACEi—Angiotensin-Converting Enzyme inhibitors; ARB—Angiotensin Receptor Blocker; IABP—intra-aortic balloon pump; MRA—Mineralocorticoid Receptor Antagonist; RRT—Renal Replacement Therapy; MV—mechanical ventilation; VIS—Vasoactive Inotropic Score. Boldface denotes statistical significance.

## Data Availability

The data presented in this study are available on request from the corresponding author.

## Abbreviations

ARDS	Acute Respiratory Distress Syndrome
CRUSADE	Can Rapid risk stratification of Unstable angina patients Suppress ADverse outcomes with Early implementation of the ACC/AHA guidelines
CS	cardiogenic shock
ECMO	extracorporeal membrane oxygenation
GRACE	Global Registry of Acute Coronary Events
IABP	intra-aortic balloon pump
ICD-10	International Classification of Diseases, 10th Revision
MI	myocardial infarction
MS	mixed shock
MV	mechanical ventilation
PCI	percutaneous coronary intervention
SCAI	Society for Cardiovascular Angiography and Interventions
SIRS	systemic inflammatory response syndrome
SOFA	Sequential Organ Failure Assessment
TIMI	Thrombolysis In Myocardial Infarction
VIS	Vasoactive Inotropic Score
